# Automatic assessment of atherosclerotic plaque features by intracoronary imaging: a scoping review

**DOI:** 10.3389/fcvm.2024.1332925

**Published:** 2024-04-29

**Authors:** Flavio Giuseppe Biccirè, Dominik Mannhart, Ryota Kakizaki, Stephan Windecker, Lorenz Räber, George C. M. Siontis

**Affiliations:** Department of Cardiology, Bern University Hospital, University of Bern, Bern, Switzerland

**Keywords:** artificial intelligence, automatic assessment, intracoronary imaging, plaque features, optical coherence tomography, intravascular ultrasound

## Abstract

**Background:**

The diagnostic performance and clinical validity of automatic intracoronary imaging (ICI) tools for atherosclerotic plaque assessment have not been systematically investigated so far.

**Methods:**

We performed a scoping review including studies on automatic tools for automatic plaque components assessment by means of optical coherence tomography (OCT) or intravascular imaging (IVUS). We summarized study characteristics and reported the specifics and diagnostic performance of developed tools.

**Results:**

Overall, 42 OCT and 26 IVUS studies fulfilling the eligibility criteria were found, with the majority published in the last 5 years (86% of the OCT and 73% of the IVUS studies). A convolutional neural network deep-learning method was applied in 71% of OCT- and 34% of IVUS-studies. Calcium was the most frequent plaque feature analyzed (26/42 of OCT and 12/26 of IVUS studies), and both modalities showed high discriminatory performance in testing sets [range of area under the curve (AUC): 0.91–0.99 for OCT and 0.89–0.98 for IVUS]. Lipid component was investigated only in OCT studies (*n* = 26, AUC: 0.82–0.86). Fibrous cap thickness or thin-cap fibroatheroma were mainly investigated in OCT studies (*n* = 8, AUC: 0.82–0.94). Plaque burden was mainly assessed in IVUS studies (*n* = 15, testing set AUC reported in one study: 0.70).

**Conclusion:**

A limited number of automatic machine learning-derived tools for ICI analysis is currently available. The majority have been developed for calcium detection for either OCT or IVUS images. The reporting of the development and validation process of automated intracoronary imaging analyses is heterogeneous and lacks critical information.

**Systematic Review Registration:**

Open Science Framework (OSF), https://osf.io/nps2b/.

**Graphical Abstract:**
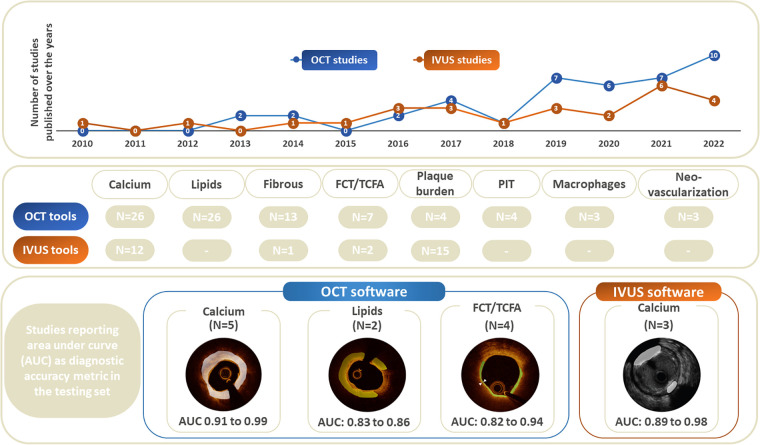
Central Illustration.

## Introduction

Since its advent in the early 1990s, intracoronary imaging (ICI) has become a mainstay clinical and research tool able to expand luminal information derived from conventional invasive coronary angiography to the description of the vascular wall components (1, 2). The two most commonly used ICI modalities, namely intravascular ultrasound (IVUS) and optical coherence tomography (OCT), are able to provide detailed intracoronary images, allowing adequate characterization of plaque components, which has been shown to be critical in percutaneous coronary interventions (2–6). Accordingly, the most recent international guidelines have upgraded their endorsement for ICI use during clinical practice (7), especially in specific settings such as left main disease and bifurcations (8, 9).

The use of ICI has also been applied to identify coronary lesions with high-risk plaque features related to future coronary events: a large plaque burden, an extensive lipid component and a thin fibrous cap (10–12).

Past studies conducted in a core laboratory setting have shown a high inter- and intra-observer reproducibility for IVUS and OCT image assessment (13–17). However, the interpretation of coronary plaque features by means of ICI in routine clinical practice remains challenging, and the use of a central core laboratory analysis has been suggested to achieve more reproducible ICI evaluation (2, 18–20). To overcome these limitations, substantial efforts have been made to develop innovative ICI build-in software able to provide an automatic assessment of coronary plaque features and guide clinical decisions. The application of automatic IC imaging analysis tools is constantly increasing (21–23). However, a lot of uncertainties persist, and, to date, the availability and diagnostic performance of these novel technologies has not been systematically investigated. Against this background, we systematically summarized the available tools for automatic evaluation of ICI modalities, compared the diagnostic accuracy, and mapped the development and validation process of those tools.

## Methods

This study is reported according to the Preferred Reporting Items for Systematic Reviews and Meta-Analyses (PRISMA) Extension for Scoping Reviews (PRISMA-ScR) (24) and synthesis without meta-analysis (SWiM) (25) statements. Patients and the public were not involved in the design, conduct, reporting, or dissemination plans of this study. Ethical approval was not required for this scoping review as primary data were not collected. The study was conducted based on a prespecified protocol, and was registered in the Open Science Framework (OSF) (https://osf.io/nps2b/).

### Diagnostic imaging modalities of interest

We included studies reporting automatic OCT or IVUS assessment as these are the most broadly used ICI modalities in daily clinical practice. The OCT technique is increasingly used and, thanks to its 10–20 microns axial resolution, enables precise visualization of the coronary wall and atherosclerotic plaque components such as calcium burden, lipid accumulation, fibrous tissue and macrophage accumulation (2). The IVUS technique offers grayscale images with an axial resolution of 80–120 *μ*m and a penetration depth of 4–8 mm. Deep penetration allows the acquisition of tomographic images of the entire coronary vessel wall, especially plaque burden (the percentage of plaque area within the entire vessel area) and calcification (2).

### Literature search strategies

We conducted a systematic review of the literature searching MEDLINE via PubMed and Embase database using a combination of the following keywords: “optical coherence tomography”, “intravascular ultrasound”, “artificial”, “intelligence”, “machine”, “learning”, “neural network”, “deep”, “learning”, “lipid*”, “calci*”, “fibrot*” or “hierarchical”. Detailed search algorithms are provided in the Supplementary Appendix Section 1. As very recent topic with most of the automatic tools only developed in the last few years, the research strategy, performed according to PRISMA guidelines, included only recent literature on the topic and limited inclusion to studies published after January 1, 2010.

### Study selection process

The study selection was performed in sequential phases. In the first phase, relevant studies in title and abstract level were obtained by combined searches of electronic databases using the above-mentioned keywords. In the second phase, potentially eligible studies were reviewed to assess the appropriateness with the study question in full text. In February 2023, two investigators (FGB and DM) independently screened titles and abstracts of identified manuscripts through the online database searches for eligibility. The same reviewers reviewed the potentially eligible studies for appropriateness and completeness in full text. The reviewers scrutinized the eligible manuscripts and extracted the required data independently. Disagreements were resolved in consensus with a third investigator (GCMS).

### Inclusion and exclusion criteria in study level

We included studies reporting development or validation of an automatic tool assessment of IVUS or OCT images. We considered eligible studies that recruited patients with coronary artery disease undergoing coronary artery wall/plaque evaluation by means of OCT or IVUS of native coronaries arteries. We included *in-vivo* and *ex-vivo* studies reporting validation and clinical application of OCT/IVUS software able to obtain an automatic assessment of native coronary wall tissue and plaque components. We included studies of any design, prospective/retrospective diagnostic studies and post-hoc analysis reporting validation of automatic ICI software compared to manual assessment, core laboratory evaluation or pathological findings (specimen). We did not consider studies that evaluated the diagnostic performance of automatic tools on stent evaluation. We also excluded studies of non-original design and experimental studies.

### Imaging features of interest

Imaging features of interest were defined as coronary wall and plaque components considered relevant in clinical practice and research. Appropriate recognition and evaluation of such features in ICI images is mandatory to succeed precision and appropriate guide the treatment strategy in patient level (2). More specific, we focused on the following features: calcium, lipid, fibrotic tissue, plaque burden, fibrous cap thickness, pathological intimal thickening, neovascularization, macrophage infiltration, calcified nodules, cholesterol crystals and microchannels. Automatic evaluation of coronary lesion with the proposed automatic tools may result in reproducible and faster ICI assessment.

### Data extraction and charting

From the main report and any accompanied material of each eligible study, we extracted the following information: first author, year of publication, recruitment period, study design (retrospective vs. prospective), funding source(s), previously published study protocol, dataset (*ad hoc* enrollment or sub-analysis of previous datasets), clinical setting, sample size, number of coronary segments evaluated), the specific imaging methods with details on imaging acquirement (OCT: domain [time vs. frequency], manufacturer, pullback speed; IVUS: imaging system, manufacturer, transducer frequency [MHz], pullback speed, applied technique to assess wall composition [i.e., grey scale or virtual histology)], applied machine learning methods for automated tool development (training), testing/validation methods (testing/external), imaging features considered (calcium, lipid, fibrotic tissue, plaque burden, fibrous cap thickness, pathological intimal thickening, neovascularization, macrophage infiltration, calcified nodules, cholesterol crystals and microchannels), FDA/EC/EMA approval of the automatic tool. We captured how the definition/diagnosis of the imaging feature(s) of interest was justified (i.e., independent evaluators). The following quantitative discriminatory metrics were extracted: sensitivity, specificity, and diagnostic accuracy (AUC).

### Data analysis and qualitative synthesis

We summarized descriptive characteristics of the studies in separate for IVUS and OCT automatic tools. We provided an overview of the available automatic tools, their characteristics and reported diagnostic performance for identification of the ICI features of interest. We evaluated the changes in diagnostic performance between the development (training and testing data set) and validation process of the automatic tools, when available. Values were considered to refer to the testing set only if clearly specified.

## Results

### Study selection process

**Figure 1:**
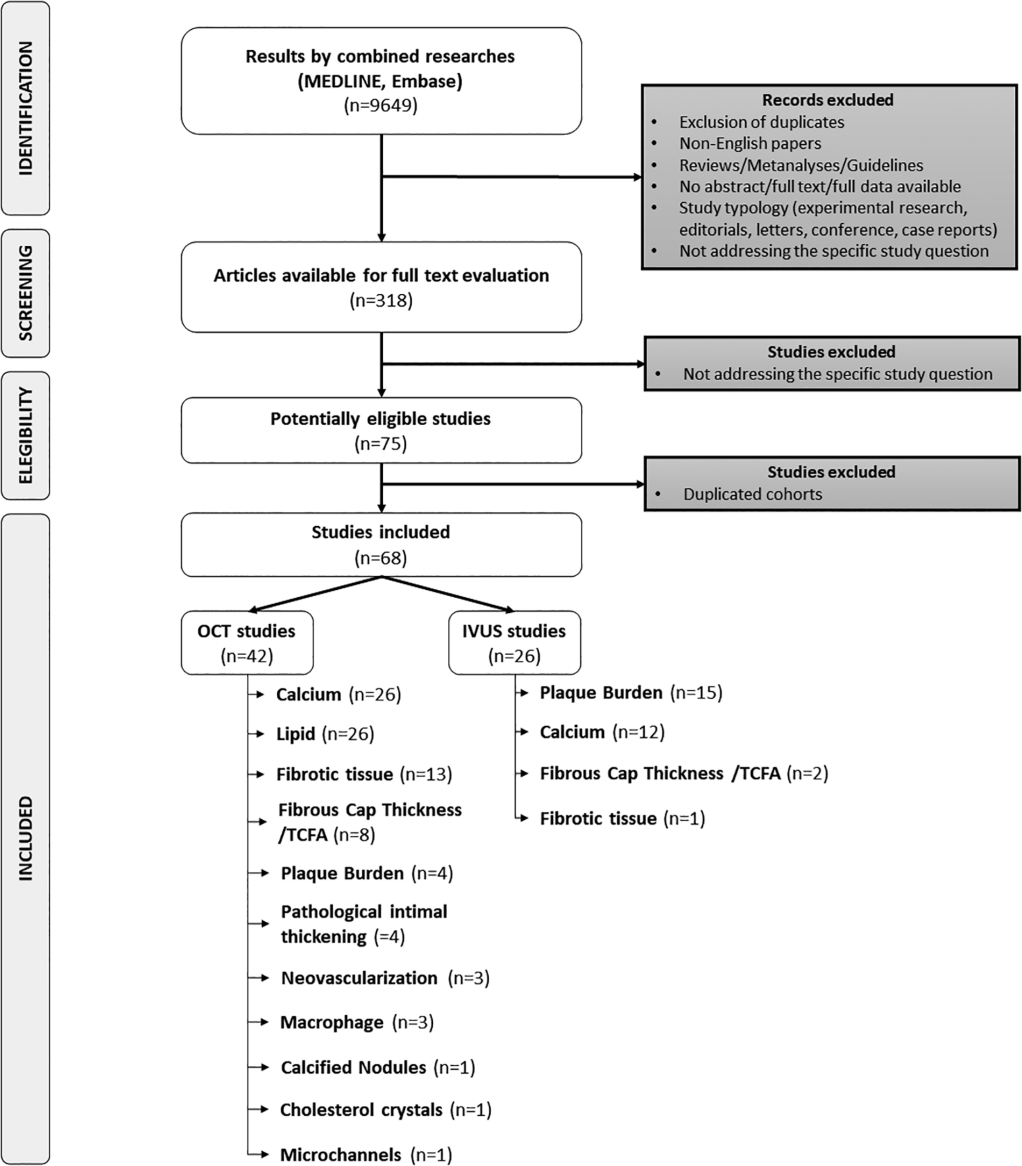
Study selection flow-chart.

The study selection flow-chart is provided in Figure 1. The literature search resulted in 9,649 citations. After screening in title and abstract level, we identified 318 potentially eligible reports, which were further evaluated in full text. Seventy-five study reports were deemed eligible. Of those, 7 reports were further excluded because of overlapping cohort populations. Finally, 68 reports were included in the scoping review (42 and 26 reports on OCT and IVUS automatic tools respectively) (Supplementary Appendix Section 2 and 3).

### Study and automatic ICI tools characteristics

Table 1 summarizes study level characteristics of studies reporting automatic evaluation of images acquired by either OCT or IVUS. Detailed characteristics for each study are provided in the Supplementary Appendix Section 2 (Supplementary Tables 1, 2).

**Table 1:** Study level characteristics of studies reporting on tools of automatic evaluation of images acquired by OCT or IVUS.

	OCT studies (*n* = 42)	IVUS studies (*n* = 26)
Study characteristics		
Year of publication, *n* (%)		
** 2010–2016**	6 (14)	7 (27)
** 2017–2023**	36 (86)	19 (73)
Study design, *n* (%)		
Prospective	1 (2)	0 (0)
Retrospective	41 (98)	26 (100)
Funding source, *n* (%)		
Industry related	0 (0)	0 (0)
Non-industry related	36 (86)	19 (73)
Both	2 (5)	0 (0)
Not available	4 (9)	7 (27)
Approval for clinical use^a^, *n* (%)		
FDA approval	0	0
CE/EMA approval	0	0
Not available	42 (100)	26 (100)
Study protocol available, *n* (%)		
Yes	15 (36)	4 (15)
No	27 (64)	22 (85)
Reference method, *n* (%)		
Manual	34 (81)	26 (100)
Histology	6 (14)	1 (4)
Both Manual and Histology	2 (5)	0
Clinical setting, *n* (%)		
CAD	34 (81)	25 (96)
Type of CAD not reported	15 (36)	14 (54)
CCS	8 (19)	7 (27)
Both ACS/CCS syndromes	8 (19)	4 (15)
Specimen (Cadaver)	6 (14)	1 (4)
Both *in-vivo* and specimen	2 (5)	0 (0)
Number of patients		
Total^b^	3,959	1,711
Median (IQR)	42 (18–68)	18 (10–42)
Number of coronary segments		
Total^c^	4,662	3,316
Median (IQR)	49 (28–83)	350 (132–557)
Artificial intelligence method, *n* (%)		
CNN	30 (71)	9 (35)
Other	12 (29)	17 (65)
Development & validation process, *n* (%)		
Training dataset	42 (100)	26 (100)
Testing dataset	38 (91)	24 (92)

ACS, acute coronary syndrome; CAD, coronary artery disease; CNN, convolutional neural network; IQR, interquartile range; CCS, chronic coronary syndromes.

^a^ Based on what is reported in the corresponding paper.

^b^ Number of individuals not available in 9 OCT studies and 2 IVUS studies.

^c^ Number of coronary segments not available in 6 OCT studies and 19 IVUS studies.

#### OCT studies

Among the 42 OCT studies, the majority were published during the period 2017 to 2022 (36 studies), with only 7 studies published between 2010 and 2016 (Table 1, Supplementary Table S1). The distribution of corresponding publications over time is illustrated in the Central Illustration. All but one study had a retrospective design, and a total of 3,959 subjects (median [IQR] of 42 [18–68]) were examined (total number of coronary segments 4,662 [49 (28–83)]. Thirty-six studies reported non-industry funding. Study protocols were available in one third of the studies. None of the automatic algorithms was accepted for clinical use at the time of the study publication. Eight studies used pathological validation to develop the automated tool. The most common machine learning method to develop the imaging analysis algorithms was the CCN method (71%). A testing dataset was available in 38 out of 42 studies.

#### IVUS studies

Of the 26 IVUS studies, 19 were published during the period 2017 to 2022 (Table 1, Central Illustration). Study level characteristics are reported in Supplementary Table S2. All had a retrospective design. Information on approval for clinical use was not provided for any of the newly developed tools. Only one study used histology as the reference method for validation, and approval for clinical use was not reported in any manuscript. A study protocol was missing for the vast majority of the studies. The median (IQR) study sample size was 18 (10–42), with median (IQR) number of assessed coronary segments of 350 (132–557). Among IVUS studies, the preferred machine learning method for developing the automatic imaging tool was other than CNN. Only in 34% of the studies, CNN was primarily applied for algorithm development. The algorithm was evaluated in a testing set in 24 out of 26 studies.

### Imaging characteristics assessed with automated ICI tools

As summarized in Table 2, calcium was the most commonly studied plaque feature (26 out of 42 OCT studies and 12 out of 26 IVUS studies). The other three plaque features examined by both OCT and IVUS modalities were plaque burden (4 and 15, respectively), fibrotic tissue (13 and 1, respectively) and fibrous cap thickness/thin cap fibroatheroma (TCFA) (7 and 2, respectively). OCT studies also reported automated tools for the assessment of lipid components (26 studies), pathologic intimal thickening (4 studies), neovascularization (3 studies), macrophages (3 studies), calcified nodules (1 study), cholesterol crystals (1 study), and microchannels (1 study).

**Table 2:** Intracoronary imaging features evaluated in individual studies.

	OCT studies (*n* = 42)	IVUS studies (*n* = 26)
Intracoronary imaging features, *n* (%)		
Calcium	26 (62)	12 (46)
Lipids	26 (62)	–
Fibrotic tissue	13 (31)	1 (4)
Fibrous cap thickness/TCFA	7 (17)	2 (8)
Plaque burden (EEM + lumen border)	4 (10)	15 (58)
Pathological intimal thickening	4 (10)	–
Neovascularitation	3 (7)	–
Macrophages	3 (7)	–
Calcified nodules	1 (2)	–
Cholesterol crystals	1 (2)	–
Microchannels	1 (2)	–

EEM, external elastic membrane border; TCFA, thin-cap fibroatheroma.

### Diagnostic performance of the automatic ICI tools

The diagnostic performance in training and testing datasets of the automated ICI tools to detect plaque components is shown in Supplementary Tables S3, S4 for OCT and IVUS, respectively.

#### Calcium

Among 26 OCT studies investigating automated calcium analysis, 10 (38%) reported diagnostic accuracy in the training set and 5 (19%) in the testing set. The discriminatory accuracy was consistently high in both sets, ranging from 0.72 to 0.98 in the training set and 0.91–0.99 in the testing set. Similarly, diagnostic accuracy in the training set (ranging from 0.90 to 0.91, 2 studies) and diagnostic accuracy in the testing set (ranging from 0.89 to 0.98, 3 studies) were consistently high in IVUS studies, although fewer studies described accuracy values. In both OCT and IVUS studies the specificity of automatic tools for automated calcium detection was higher than sensitivity (Supplementary Tables S3, S4, respectively*)*.

#### Lipids

Of 26 studies investigating automatic tools to detect lipid content on OCT images, 9 reported the diagnostic accuracy in the training set (ranging from 0.79 to 0.99) and only 2 described the diagnostic accuracy in the testing set (0.82 and 0.86) (Supplementary Table S3). IVUS studies reporting automatic tools for lipid evaluation were not found.

#### Fibrotic tissue

Overall, 5 out of 13 OCT studies investigating automatic fibrotic tissue detection reported the diagnostic accuracy in the training set (ranging from 0.85 to 0.96), and one with testing set (0.96). Only one IVUS study explored automatic fibrotic tissue detection, without reporting the accuracy achieved by the method.

#### Plaque burden

Plaque burden was investigated in 4 OCT studies. Among them, only one reported diagnostic accuracy in the training set (0.92), and no one in the testing set. The number of IVUS studies describing automatic tools for plaque burden was higher (*n* = 15). However, the diagnostic accuracy was reported only in one study (testing set 0.70).

#### Fibrous cap thickness

Among 8 OCT studies investigating automatic assessment of fibrous cap thickness or TCFA, 3 (38%) reported diagnostic accuracy in the training set and 4 (50%) in the testing set. The discriminatory ability was consistently high in both sets, ranging from 0.81 to 0.93 in the training sets and 0.82–0.94 in the testing set. Two IVUS studies described the diagnostic accuracy for TCFA in the training set and one in a testing set. Bae et al. (26) used a 200 µm threshold (due to the IVUS resolution) and trained IVUS against OCT images, reporting a diagnostic accuracy of 0.80 in the training set and 0.82 in the testing set for IVUS to detect TCFA <200 µm. Jun et al. (27) used OCT and IVUS images in a common pre-processing to train IVUS images in recognized OCT-detected TCFA <65 µm. By training the CNN classifier with an augmented IVUS image, the tool increased considerably the discriminatory accuracy to 0.91.

#### Other features

Other plaque features were investigated only in OCT studies: one study reported diagnostic accuracy for pathological intimal thickening (0.85 in the testing set), 1 study reported diagnostic accuracy for neovascularization in training set (0.90) and 2 in testing set (0.90 and 0.99), 1 study reported diagnostic accuracy for macrophage detection (0.88 in the training set) and 1 study reported diagnostic accuracy for calcified nodules (0.91 in both training and testing set).

## Discussion

### Summary of evidence

Tools for automatic real-time imaging analysis acquired by ICI imaging methods are increasingly applied to improve the diagnostic performance. Although such machine learning-based tools have been introduced with high expectations, their clinical applications are still limited. Our evaluation showed that:

- A limited number of automatic machine learning-based tools for ICI analysis is currently available, without proven clinical validity.
- The majority have been developed for calcium detection for either OCT or IVUS images, for assessment of lipid and FCT/TFA content on OCT images, and plaque burden on IVUS images.
- The reporting of development and clinical validation process of these tools is lacking critical information.

Appropriate interpretation of medical images can be challenging, time-consuming and is associated with high inter-observer variability (28). Against this background, researchers and physicians have recently started benefiting from computer-assisted imaging evaluations (29). In our scoping review, we found that studies investigating automatic ICI tools have considerably increased over time, but still the number of available tools are limited with no proven clinical validity. In addition, the vast majority of them are non-industry funded and investigator-initiated, reflecting the clinical need and interest of clinicians to get access to such tools in routine clinical practice.

#### Development of automatic tools ICI analysis

Plaque tissue characterization involves the identification and classification of different tissue layers. Automation of the tissue characterization process requires a machine learning-based software tool to provide a real-time analysis able to help physicians in routine clinical practice. Generally, machine learning-based algorithms require the use of a gold standard as prior information, and based on these prior labels (classes) (supervised approaches), training coefficients are estimated using the training image dataset. Among machine learning techniques, convolutional neural networks (CNNs), a deep learning-based technique, has gained a lot of attention in medical imaging mainly due to the ability to extract more high-level features (29). We found that CNN was the most commonly used method to develop the imaging analysis algorithm in OCT (71%) but not in IVUS studies (34%). This is probably related to the increasing number of automated OCT automatic tools in the recent years, while most IVUS tools were developed more than 5 years ago. When considering IVUS studies published in the last 3 years, most of them applied CCNs.

#### Imaging characteristics assessed with automated ICI tools

In our study, we found an increasing use of automated OCT tools, especially for calcium (62%), lipids (62%), fibrous tissue (31%) and fibrous cap thickness (19%). Conversely, IVUS studies have increased less in recent years, and focused more on the automatic assessment of plaque burden (58%), and on calcium (46%) (Graphical Abstract).

**Figure 2:**
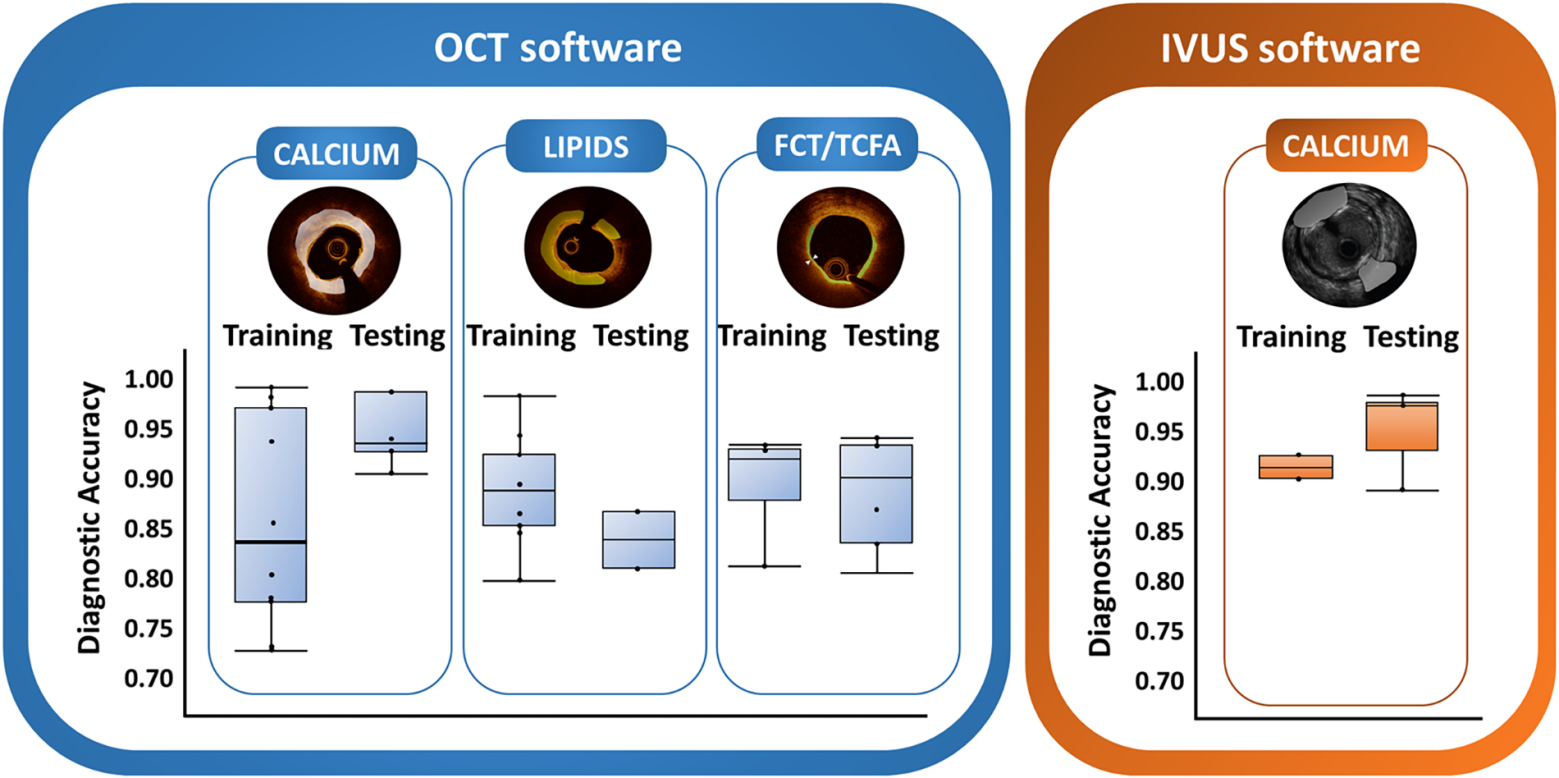
Distribution of discriminatory performance (area under the curve) of automatic tools in training and testing datasets for OCT and IVUS.

Previous data have shown that the detection of plaque calcium burden can identify vessels at higher risk of suboptimal stent implantation in which more aggressive debulking techniques can be of benefit (3). As the presence of calcium can be detected with good accuracy at both OCT and IVUS manual assessment, it is not surprising that the vast majority of both OCT and IVUS studies developed algorithms to automatically detect calcium. The accuracy described with both techniques was high and consistent (0.72–0.98 with OCT and 0.89–0.98 with IVUS), although OCT studies reported more frequently accuracy metrics than IVUS (Figure 2). Of note, the accuracy shown by IVUS techniques was not dependent on the frequency of image acquisition (30). Collectively, these data suggest that automated calcium assessment stands out as a tool suitable for early integration into clinical practice.

A large lipid core and a TCFA are well-described characteristic of plaque vulnerability and represent important information during PCI to achieve adequate lesion coverage and safe landing zones (12, 31). Nonetheless, routine manual assessment of lipid components and fibrous cap thickness remains challenging and study investigating the inter-observer variability showed controversial results (19, 20). In our study, lipid and fibrous cap thickness automatic measurements represented the second and third most frequent OCT features under investigation, respectively. A cap thickness of 65 *μ*m was used to differentiate TCFA and fibroatheroma in OCT studies (32–34). OCT software showed high accuracy >0.80–0.85 for both lipid and FCT/TCFA automatic evaluation. However, only two studies described the accuracy for automatic lipid detection and four for automatic cap thickness quantification (22, 32–36). No IVUS software was developed to detect intra-plaque lipid accumulation. It is well acknowledged that greyscale IVUS is affected by a low accuracy in recognizing hypoechogenic tissues like lipids. This limitation has been overcome with the introduction of the combination catheter IVUS-near-infrared spectroscopy (IVUS-NIRS), a modality that can recognize intraplaque cholesterol accumulation with chemograms (37). A few studies investigated tools to automatically detect FCT/TCFA on IVUS images. However, despite good accuracy, the cut-off of 65 *μ*m could not be applied in IVUS studies due to the lower resolution of this technique. The 200 µm IVUS threshold used by Bae et al. (26) to identify IVUS-derived TCFA has not been clinically validated for clinical outcomes and is far from the plaque vulnerability thresholds shown by pathologic studies (38). A higher number of studies investigated the automatic quantification of plaque burden at IVUS images. Even though this could be the most valuable application of machine learning-based tools in IVUS images, only one reported the diagnostic performance in the testing dataset, which was moderate (diagnostic accuracy of 0.70) (26).

#### Clinical application and future considerations

Distinct steps of development (training and testing), validation and clinical evaluation of machine learning-based algorithms for diagnosis or prediction purposes have been established (39–42). In the case of machine learning-derived tools either as standalone medical device software or embedded within an intracoronary imaging modality, there are additional challenges to be considered (43). A few studies have already reported effective applications of automatic ICI evaluation in a clinical context. In 103 patients undergoing high-intensity statin therapy, Blanco et al. showed similar results between manual annotation and machine learning-based evaluation in detecting favorable changes in percent of atheroma volume (44). The potential usefulness of a machine learning-based approach for the study of plaque vulnerability was recently shown in a CLIMA substudy investigating an OCT-derived lipid core burden index (OCT-LCBI). A large lipid accumulation detected by a CNN algorithm (maximum OCT-LCBI in 4 mm segment ≥400) was significantly associated with a thin fibrous cap <75 µm and future cardiac events (45). Similarly, Niioka et al. reported that an OCT-derived TCFA, diagnosed by a CNN-based algorithm, was independently associated with clinical events (36). Recently, integrated dual-modality imaging systems combining OCT with NIRS or near-infrared fluorescence have been explored in *ex-vivo* and experimental studies to implement chemogram-based lipid detection in OCT catheters (46–48). However, definitive data and clinical application of these techniques are lacking to date (49, 50).

## Limitations

Our study has several limitations. First, we investigated only diagnostic accuracy, without reporting other metrics such as calibration and correlation coefficients. However, the included studies were characterized by inadequate and considerably heterogeneous reporting, which did not allow us to summarize other quantitative metrics of interest. This also suggests that the specific field requires the establishment of standardized approaches on how to develop and report such machine learning-derived algorithms for ICI evaluation. Second, due to the heterogeneous reporting in diagnostic performance metrics, we were not able to derive any conclusions about the comparative performance among these newly developed machine learning-based tools and across the distinct steps of training/testing. Third, we included only machine learning-based tools described in peer-reviewed publications. This approach may have excluded tools which are described only on companies' websites or preprint platforms.

## Conclusions

A limited number of automatic machine learning-derived tools for ICI analysis is currently available, without proven clinical validity. The majority have been developed for calcium detection for either OCT or IVUS images. The reporting of the development and validation process of automated intracoronary imaging analyses is heterogeneous and lacks critical detail.

## Data Availability

The original contributions presented in the study are included in the article/Supplementary Material, further inquiries can be directed to the corresponding author.
