# Supplementary material for: Automatic assessment of atherosclerotic plaque features by intracoronary imaging: a scoping review
Source: Front Cardiovasc Med. 2024 Apr 29;11:1332925. doi: 10.3389/fcvm.2024.1332925 (PMC11090039; doi:10.3389/fcvm.2024.1332925)
Supplement: Supplementary file 1 [file Datasheet1.pdf]

Automatic Assessment of Atherosclerotic Plaque Features by Intracoronary Imaging: A Scoping Review.

Appendix

Contents

Appendix Section 1 .... **1**

Detailed literature search strategy. 1

Appendix Section 2 .... **3**

Supplementary Table 1. Study-level characteristics for OCT-related software. 3

Supplementary Table 2. Study-level characteristics for IVUS-related software. 10

Appendix Section 3 .... **17**

Supplementary Table 3. Quantitative metrics for diagnostic performance of automatic OCT tools. 17

Supplementary table 4. Quantitative metrics for diagnostic performance of automatic IVUS tools. 35

Appendix Section 1

Detailed literature search strategy.

Ovid MEDLINE(R) ALL <2010 to February 1, 2023>

1 exp coronary artery disease/
2 (optical coherence tomography* or intravascular ultrasound* or coronary* or atheroscler* or plaqu*).tw,kf.
3 (OCT or IVUS or IVOCT or AI or CAD or CHD).ti,kf.
4 or/1-3
5 exp Artificial Intelligence/
6 Machine Learning/
7 exp Deep Learning/
8 exp Natural Language Processing/
9 exp neural networks, computer/
10 (artificial adj1 intelligence).tw,kf.
11 ((deep or machine or hierarchical) adj2 learning).tw,kf.
12 (vector adj3 machine).tw,kf.
13 ((deep or convolutional or neural) adj3 network*).tw,kf.
14 (natural language adj2 process*).tw,kf.
15 ((supervised or unsupervised) adj2 machine learning).tw,kf.
16 AI.ti,kf.
17 or/5-16
18 systematic review.pt.
19 review.pt.
20 meta analysis.pt.
21 case report.pt.
22 letter.pt.
23 editorial.pt.
24 clinical conference.pt.
25 or/18-24
26 4 and 17
27 26 not 25
28 limit 27 to yr="2010-2023"

Embase ALL <2010 to February 1, 2023>

1 'coronary artery disease'
2 'optical coherence tomography' OR 'intravascular ultrasound'
3 'artificial intelligence'
4 'convolutional neural network'
5 'machine learning'
6 'deep learning'
7 'neural networks'
8 'automatic'
9 'systematic review' OR 'review'
10 'meta analysis'
11 'letter'

12 'case report'
13 'editorial'
14 'conference'
15 #1 OR #2
16 #3 OR #4 OR #5 OR #6 OR #7 OR #8
17 #15 AND #16
18 #9 OR #10 OR #11 OR #12 OR #13 OR #14
19 #17 NOT #18 AND [2010-2023]/py
20 limit #19 to yr="2010-2023"

Appendix Section 2

Supplementary Table 1. Study-level characteristics for OCT-related software.

First Author (PMID)	Year of publication (PMID or DOI)	Study design	Funding sources	Previously published study protocol	Validation	Clinical setting (ACS, CCS)	Number of items	Pullbacks or segments	Frames/images total	Methods/AI	OCT Domain	OCT catheter company	Pullback speed (mm/s)
**Ughi et al. (23847728)**	2013	R	Research Foundation Flanders FWO, KU Leuven IOF grant and FWO foundation (BE)	yes	manual		49	49	64	supervised random forest (RF) - attenuation	fourier domain	St. Jude Medical	20
**Athanasiou LS et al. (24109966)**	2013	R	European Regional Development Fund and Greek national funds	-	manual		10		27	k-means clustering method	frequency domain	LightLab Imaging	-
**Athanasiou LS et al. (24525828)**	2014	R	-	-	manual	Routine Coronary Angiography	22	27	556	random forests (RF)].	fourier domain	Lightlab Imaging	-
**Celi S et al. (25077844)**	2014	R	-	-	manual	Routine Coronary Angiography	10	10	2800	Image binarization/ Otsu thresholding method/Savitzky-Golay	fourier domain	LightLab Imaging	20

										algorithm			
**Rico-Jimenez et al. (27867716)**	2016	R	National Heart, Lung, and Blood Institute and Institute of Biomedical Imaging and Bioengineering (US)		histology	Cadaver		57	513	Linear discriminant analysis (LDA)	frequency domain	na	
**Gnanadesigan M et al. (27998841)**	2016	P	Dutch Heart Foundation	yes	manual	PCI for stable/unstable angina pectoris or acute MI	85	98		index of plaque attenuation by single scattering model	na	St. Jude Medical	20
**Gnanadesigan et al. (27620900)**	2017	R	Netherlands Heart Foundation Grant, Heart Research UK		histology	Random cadaver hearts	6			index of plaque attenuation (IPA) (optical attenuation coefficient)	na	St. Jude Medical	20
**Liu et al. (28901053)**	2017	R	China Scholarship Council		histology		2	2	135	Attenuation, Backscatter intensity	frequency domain	Terumo Lunawave	20
**Zhou P et al. (29036125)**	2017	R	National 973 Basic Research Program of China, National Natural Science Foundation of China and Australian Research Council	-	manual	-	9	10	-	random forests (RF)	fourier domain	Lightlab Inc.	-

**Mengdi Xu et al. (29060164)**	2017	R	-	-	manual	-	18	18	360	CNN	frequency domain	Terumo	-
**Kolluru et al. (30525060)**	2018	R	National Heart, Lung, and Blood Institute (US)	yes	manual		48	48	4469	CNN/ANN	frequency domain	St. Jude Medical	36
**Gessert et al. (30130180)**	2019	R	-	-	manual	Routine Coronary Angiography	49	49	4000	CNN	na	St. Jude Medical Ilumien OPTIS	-
**Liu R et al. (31535296)**	2019	R	Fundamental Research Funds for the Central Universities, Chongqing Foundation and Advanced Research Project, Science and Technology Research Program of Chongqing Municipal Education Commission, Sichuan Science and Technology Progam and Entrepreneurship and Innovation Program for Chongqing Overseas Returned Scholars	-	manual	-	-	-	2300	CNN	-	-	-
**Lee et al. (31853413)**	2019	R	National Institutes of Health (US)	yes	manual	-	55	57	4892	CNN	frequency domain	St. Jude Medical	36
**Rico-Jimenez et al. (31604172)**	2019	R	National Institute of Health (US)	-	histology	Cadaver	-	28	252	normalized-intensity standard deviation	na	na	-

**Gharaibeh et al. (31903407**	2019)	R	National Heart, Lung, and Blood Institute US National Institute of Health. Choose Ohio First Scholarship	-	manual	-	-	34	2640	CNN	frequency domain	St. Jude Medical Ilumien OPTIS	36
**Athanasiou et al. (DOI: 10.1117/12.2513078)**	2019	R	NVIDIA and US Natonal Institute of Health	-	manual	-	28	28	700	CNN	frequency domain	St. Jude Medical	-
**Yang et al. (https://doi.org/10.1016/j.bbe.2019.06.006)**	2019	R	National Natural Science Foundation of China and Natural Science Foundation of Hebei Province	yes	manual	-	9	20	1700	Hard example mining (HEM) training strategy to train SVM classifier	fourier domain	St. Jude Medical	-
**Min et al. (31718998)**	2020	R	Korea Healthcare Technology R&D Project, Ministry for Health & Welfare Affairs of Korea and Ministry of Science and ICT	-	manual	stable and unstable angina	602	602	45400	CNN	na	St. Jude Medical	20
**Gerbaud E et al. (31326995)**	2020	R	National Institute of Health (US)	-	manual	elective percutaneous coronary intervention	42	96	200	EEL-enhancement algorithm	frequency domain	na	20
**Lee et al. (32054895)**	2020	R	National Heart, Lung, and Blood Institute (US)	yes	manual	-	49	49	6556	CNN	frequency domain	St. Jude Medical	36

**He et al. (32914606)**	2020	R	National Nature Science Foundation of China and ARC	-	manual	-	24	24	4860	CNN	na	St. Jude Medical	-
**Lee et al. (33598377)**	2020	R	National Heart, Lung, and Blood Instituted (US)	yes	histology+ manual	-	68	68	8231 in-vivo/4320 ex vivo	3D CNN	frequency domain	St. Jude Medical	36
**Lee et al. (35291576)**	2020	R	National Heart, Lung, and Blood Institute (US)	-	manual	-	-	48	4292	CNN+CRF	frequency domain	St. Jude Medical	36
**Chu et al. (33528359)**	2021	R	National Key Research and Development Program of China, Science Foundation Ireland	-	manual	ACS, Stable Angina, Unstable Angina, Silent ischaemia	391	509	11673	CNN	frequency domain	Abbott C7-XR™ or OPTIS™	-
**Abdolmanafi et al. (33914917)**	2021	R	BoBeau Coeur|Fondation CHU Ste-Justine, Montreal	yes	manual	-	-	41	8200	CNN	frequency domain	St.Jude Medical FD-OCT	20
**Avital et al. (34050203)**	2021	R	-	-	manual	Routine Coronary Angiography	-	-	8000	CNN	na	na	-
**Shibutani et al. (34126504)**	2021	R	JSPS KAKENHI (Japan), Japan Arteriosclerosis Prevention Fund	yes	histology	Cadaver	45	83	1103	CNN	frequency domain	Terumo Lunawave	20
**Yin et al. (34222368)**	2021	R	National Natural Science Foundation of China and Australian Research Council	-	manual	-	31	31	2000	CNN	na	St. Jude Medical	-
**Isidori et al. (34292435)**	2021	R	Centro per la Lotta contro l'Infarto- CLI–Fondazione Onlus	-	manual	STEMI/NSTEMI/Unstable Angina/Stable Angina	103	103	4500	CNN	frequency domain	St Jude Medical	20
**Holmberg et al.**	2021	R	German Cardiac Society and European Research Council	-	manual+histology	Routine Coronary Angiography	58	58	3568	CNN	na	LightLab Imaging Inc	-

**(34970608)**													
**Wu X et al. (35061247)**	2022	R	National Key Research and Development Program of China, National Natural Science Foundation of China, CAS Youth Inoovation Promotion Association, CAS Key Technology Talent Program, Natural Science Foundation of Beijing City and The Project of High-Level Talents Team Introduction in Zhuhai City	-	manual	-	70	70	1950	Structure attention co-training neural network	na	St.Jude Medical	-
**Niioka H et al. (35982217)**	2022	R	Japan Agency for Medical Research and Development	-	manual	OCT imaging of non culprit lesions	1791	1791	46120	CNN	frequency domain	Abbott Vascular	36
**Sun H et al. (35991920)**	2022	R	National Natural Science Foundation of China, Sichuan Science and Technology Program, the Fundamental Research Funds for the Central Universities and Newton Fund	-	manual	ACS	83	83	29914	CNN	na	Abbott Vascular	-
**Rico-Jimenez JJ et al. (36307914)**	2022	R	National Institutes of Health, Cancer Prevention and Research Institute of Texas and Oklahome Tobacco Settlement Endowment Trust	-	histology	-	5	10	98	CNN	-	-	5
**Lee J et al. (36354559)**	2022	R	National Heart, Lung, and Blood Institute and American Heart Association	yes	manual	Stable Angina with documented ischemia or ACS	79	122	8403	CNN	frequency domain	Abbott Vascular	36

**Olender et al. et al. (35272217)**	2022	R	MathWorks, EPFL WISH Foundation (Switzerland) and the Knut and Alice Wallenberg Foundation (Sweden)	yes	manual	Routine Coronary Angiography	21	-	-	CNN	frequency domain	St. Jude Medical	-
**Lee J et al. (36465096)**	2022	R	National Heart, Lung, and Blood Institute and American Heart Association	yes	manual	-	41	48	3450	CNN	frequency domain	St. Jude Medical	36
**Lee J et al. (36509806)**	2022	R	National Heart, Lung, and Blood Institute and American Heart Association	yes	manual	stable angina and documented ischemia or ACS	41	77	4360	CNN	frequency domain	St. Jude Medical	36
**Huang J et al. (36519717)**	2022	R	Natural Science Foundation of China and Science Foundation Ireland Research Professorship	yes	manual	5-year follow-up after implation of secong Gen Absorb bioresorbable vascular scaffold (Abbott)	15	64	-	CNN	frequency domain	Pulse Medical	-
**Chen T et al. (36434330)**	2022	R	Natural Science Foundation of Hei Longjiang Province	-	manual	-	-	150	4824	CNN	frequency domain	-	-
**Shi P et al. (36635865)**	2023	R	National Key Research and Development Program of China and National Natural Science of Foundation of China	yes	manual	-	-	-	2300	CNN	-	-	-

Supplementary Table 2. Study-level characteristics for IVUS-related software.

First Author (PMID)	Year of publication	Study design	Funding sources	Previously published study protocol	Validation	Clinical setting (ACS, CCS)	Number of items	Pullbacks or segments	Frames/images total	Methods/ AI	Company	transducer frequency (MHz)	pullback speed (mm/s)
**Zhang Q et al. (19900745)**	2010	R	National Basic Research Program of China, Shanghai Leading AcademicDiscipline Project and InnovationFund for Fudan Graduate Students	-	M	-	11	-	86	Active contour and contourlet transform	Boston Scientific	30 and 40	-
**Vard A et al. (22415899)**	2012	R	na	-	M	known CAD	5	-	1500	active contour through normalized cumulative short-term autocorrelation	Volcano Therapeutics	30	0.5
**Gao et al. (25372784)**	2014	R	Fund for Low-cost Health-care Technologie in China, Enhancing Program of Key Laboratories of Shenzhen City, National High-tech R and D Program, National Natural Science Foundation of China, Inovation funding in Shenzhen	-	M	-	8	-	996	Rayleigh mixture model, Markov random fields and belief propagation algorithm	Volcano Corp.	20	0.5

**Gao Z et al. (25922134)**	2015	R	Guangdong Innovation Research Team Fund for Low-Cost Health-Care Technologies in China, the National High-Tech R & D Program, the Guangzhou Science and Technology Planning Project, Key Lab for Health Informatics of the Chinese Academy of Sciences, the Enhancing Program of Key Laboratories of Shenzhen City the innovation funding of Shenzhen government, the Shenzhen Innovation Funding and the National Natural Science Foundation of China	-	M	CAD	6	-	337	multiclassifier with k-means clustering and 2-D Otsu method	Volcano Corporation	20	0.5
**Araki T et al. (26643081)**	2016	R	-	1	M	stable angi pectoris	15	-	30600	K-means, Fuzzy c-Means and Hidden Markov Random Field.	Boston Scientific	40	0.5
**Banchhor SK et al. (27480747)**	2016	R	-	-	M	stable angina pectoris	19	-	38760	threshold, FCM, K-means, and HMRF	Boston Scientific	40	0.5

**Lo Vercio L et al. (26811082)**	2016	R	National Agency for Science and Technology Promotion (ANPCyT, Argentina)	-	M	-	-	-	149	support vector machines	Volcano Corporation	20	-
**Banchhor SK et al. (28390284)**	2017	R	-	-	M	-	19	-	40090	soft pixel classification (Threshold, FCM, K-means, and HMRF)	Boston Scientific	40	0.5
**Su S et al. (28062170)**	2017	R	National Natural ScienceFoundation of China, Guangdong Image-guided Therapy Innovation Team and national Keyresearch and Development Program of China	-	M	-	4	-	461	ANN	Volcano Corporation	20	0.5
**Zakeri FS et al. (28372789)**	2017	R	-	-	M	-	6	-	-	Automatic contour detection, sparse representation framework, dynamic directional gradient vector field	Volcano Therapeutics	30	0.55
**Faraji M et al. (29241056)**	2018	R	-	-	M	-	10	10	326	Extremal Regions of Extremum Levels (EREL)	Volcano Corporation	20	-

**Bae Y et al. (31130215)**	2019	R	Korea Healthcare Technology R&D Project, Ministry of Science and ICT and Asan Institute for Life Sciences (Korea)	-	OCT-TCFAs	stable and unstabl e AP	517	517	40908	artificial neural network (ANN), support vector machine (SVM), and naïve Bayes	Boston Scientific	40	0.5
**Jun TJ et al. (30426362)**	2019	R	International Research and Development Program of the National Research Foundation of Korea, Ministry of Science, ICT and Future Planning of Korea	1	M (OCT)	stable or unstabl e ap	100	-	12325	feed-forward neural network (FNN), K-nearest neighbor (KNN), random forest (RF), and CNN	Boston Scientific	40	0.5
**Lo Vercio L et al. (31319939)**	2019	R	FONCYT - ANPCYT of Argentina and CONICET postdoctoral grant	-	M	-	10	-	435	support vector machine (SVM) and random forests	Volcano Corporati on	20	-
**Huang Y et al. (31978806)**	2020	R	National Key Research and Develop- ment Program of China	-	M	-	35	-	812	select - P - MSER (maximally stable extremal region)	Boston Scientific	40	-
**Liu S et al. (32636052)**	2020	R	-	-	M	routine clinical care	104	105	-	support vector	Infraredx	40	0.5

										machine (SVM)			
**Li et al. (34351974)**	2021	R	Show Chwan Memorial Hospital, National Health Research Institutes and Ministry of Science and Technology, Taiwan	-	M	-	18	-	713	Deep CNN	Philips Volcano, Inc.	20	-
**Shinohara et al. (33460377)**	2020	R	-	-	M	stable AP	24	-	3738	Deep neural network	AltaView	60	3
**Bajaj R et al. (34153412)**	2021	R	British Heart Foundation, University College London Biomedical Resource Centre, Rosetrees Trust and Barts NIHR Biomedical Research Centre	1	M	stable ap	65	-	26211	CNN	Infraredx	50	0.5
**Cho H et al. (33831671)**	2021	R	Korea Healthcare Technology R&D Project, Ministry for Health & Welfare Affairs, Ministry of Science and ICT and Asan Institute for Life Sciences Grant, Korea	-	M	stable or unstabl e ap	598	598	137989	CNN	Boston Scientific	40	0.5
**Dong L et al. (33549115)**	2021	R	National Natural Science Foundation of China, Zhejiang Provincial Public Welfare Technology Research Project,Zhejiang Provincial key research and development plan,	-	M	-	30	-	675	CNN	Boston Scientific	40	-

			the Major projects in Wenzhou of China and Scientific research project of Zhejiang Education Department										
**Nishi T et al. (33741429)**	2021	R	UeharaMemorial Foundation	-	M	-	11	1576	45449	CNN	Boston Scientific (40Mhz), Volcano Corporation (45 MHz) and Terumo (40Mhz)	40 and 45	0.5
**Bajaj et al. (35196627)**	2022	R	Queen Mary University of London and Barts NIHR Biomedical Research Centre	-	H	-	15	-	262	Machine learning (j48)	Infraredx	40	0.5
**Zhu et al. (35861418)**	2022	R	Henan Science and Technology Development Plan 2020, 2019 Maker Space Incubation Project of Zhengzhou University of Light Industry, Key Scientific Research Projects of Colleges and Universities in Henan Province, National Natural Science Foundation of China, China Postdoctoral Science Foundation, Pilot Project for Disruptive Technology	-	M	-	18	-	1746	Deep neural network	Volcano Corporation	-	0.5

			and China National Center for Biotechnology Development										
**Blanco PJ et al. (34670148)**	2022	R	Brazilian agencies CNPq and FAPESP and Argentinean agency ANPCyT	1	M	-	63	160	-	multi-frame CNN	Volcano Corporation	20	0.5
**Du H et al. (34974233)**	2022	R	National Natural Science Foundation of China, Guangdong Provincial Key Laboratory of Medical Image Processing and Shanghai Science and Technology Commision	-	M	-	-	350	12560	FCN (fully convolutional networks)	Boston Sci-entific,	40	0.5

Appendix Section 3

Supplementary Table 3. Quantitative metrics for diagnostic performance of automatic OCT tools.

First Author (PMID/DOI)	Training set			Testing set		
	Sensitivity	Specificity	Diagnostic Accuracy	Sensitivity	Specificity	Diagostic accuracy
Calcium						
**Ughi et al. (23847728)**	-	-	0.721	-	-	
**Athanasiou LS et al. (24109966)**	-	-	-	-	-	-
**Athanasiou LS et al. (24525828)**	0.81	-	-		-	-
**Celi S et al. (25077844)**	-	-	-	-	-	-
**Rico-Jimenez et al. (27867716)**	-	-	-	-	-	-
**Gnanadesigan M et al. (27998841)**	-	-	-	-	-	-
**Gnanadesigan et al. (27620900)**	-	-	-	-	-	-
**Liu et al. (28901053)**	-	-	-	-	-	-
**Zhou P et al. (29036125)**	-	-	>0.8	-	-	
**Mengdi Xu et al. (29060164)**	-	-	-	-		
**Kolluru et al. (30525060)**	0.8	0.95	0.777			
**Gessert et al. (30130180)**	-	-	0.78			
**Liu R et al. (31535296)**	-	-	-	-	-	-
**Lee et al. (31853413)**	0.858	0.936	-	0.851	0.942	-
**Rico-Jimenez et al. (31604172)**		-	-	-		
**Gharaibeh et al. (31903407**	0.810	-	-	0.85		-
**Athanasiou et al. (DOI: 10.1117/12.2513078)**	-	-	0.723	-	-	
**Yang et al. (https://doi.org/10.1016/j.bbe.2019.06.006)**	-	-	0.972	-	-	
**Min et al. (31718998)**	-	-	-	-	-	-
**Gerbaud E et al. (31326995)**	-	-	-	-	-	-

**Lee et al. (32054895)**	0.912	0.962	-			-
**He et al. (32914606)**	-	-	-	-	-	-
**Lee et al. (33598377)**	0.977	0.877	-	-	-	-
**Lee et al. (35291576)**	0.824	0.925	-			
**Chu et al. (33528359)**	-	-	0.855	-	-	-
**Abdolmanafi et al. (33914917)**	0.98	0.99	0.99	0.98	0.99	0.99
**Avital et al. (34050203)**	-	-	-	-	-	0.99
**Shibutani et al. (34126504)**	-	-	-	0.78	0.88	0.91
**Yin et al. (34222368)**	0.87	0.92	-			
**Isidori et al. (34292435)**	0.72	0.99	0.98	0.69	0.97	0.93
**Holmberg et al. (34970608)**	-	-	-	-	-	-
**Wu X et al. (35061247)**	-	-	-	-		
**Niioka H et al. (35982217)**	-	-	-	-	-	-
**Sun H et al. (35991920)**	-	-	-	-	-	-
**Rico-Jimenez JJ et al. (36307914)**	-	-	-	-		
**Lee J et al. (36354559)**	-	-	-	-	-	-
**Olender et al. et al. (35272217)**	0.659	0.678	-	0.659	0.678	-
**Lee J et al. (36465096)**	-	-	-	-		
**Lee J et al. (36509806)**	-	-	-	-	-	-
**Huang J et al. (36519717)**	-	-	-	-	-	-
**Chen T et al. (36434330)**	-	-	0.935	-	-	0.935
**Shi P et al. (36635865)**	-	-	-	-	-	-
**Lipids**						
**Ughi et al. (23847728)**	-	-	0.795	-	-	
**Athanasiou LS et al. (24109966)**	-	-	-	-	-	-
**Athanasiou LS et al. (24525828)**	0.71	-	-		-	-
**Celi S et al. (25077844)**	-	-	-	-	-	-

**Rico-Jimenez et al. (27867716)**	0.864	0.846	0.856	0.864	0.846	0.856
**Gnanadesigan M et al. (27998841)**	-	-	-	-	-	-
**Gnanadesigan et al. (27620900)**	-	-	-	-	-	-
**Liu et al. (28901053)**	-	-	-	-	-	-
**Zhou P et al. (29036125)**	-	-	0.915	-	-	
**Mengdi Xu et al. (29060164)**	-	-	-	-		-
**Kolluru et al. (30525060)**	0.85-(CNN)	0.92-(CNN)	0.865			
**Gessert et al. (30130180)**	-	-	0.848			
**Liu R et al. (31535296)**	-	-	-	-	-	-
**Lee et al. (31853413)**	0.887	0.883	-	0.874	0.895	-
**Rico-Jimenez et al. (31604172)**		-	-	-		
**Gharaibeh et al. (31903407**	-	-	-	-	-	-
**Athanasiou et al. (DOI: 10.1117/12.2513078)**	-	-	0.930	-	-	
**Yang et al. (https://doi.org/10.1016/j.bbe.2019.06.006)**	-	-	0.992	-	-	
**Min et al. (31718998)**	-	-	-	-	-	-
**Gerbaud E et al. (31326995)**	-	-	-	-	-	-
**Lee et al. (32054895)**	0.848	0.978	-			-
**He et al. (32914606)**	-	-	-	-	-	-
**Lee et al. (33598377)**	-	-	-	-	-	-
**Lee et al. (35291576)**	0.822	0.908	-			
**Chu et al. (33528359)**	-	-	-	-	-	-
**Abdolmanafi et al. (33914917)**	-	-	-	-	-	-
**Avital et al. (34050203)**	-	-	-	-	-	-
**Shibutani et al. (34126504)**	-	-	-	-	-	-
**Yin et al. (34222368)**	0.81	0.96	-			
**Isidori et al. (34292435)**	0.75	0.98	0.95	0.67	0.85	0.82
**Holmberg et al. (34970608)**	-	-	-	-	-	-

**Wu X et al. (35061247)**	-	-	-	-		
**Niioka H et al. (35982217)**	-	-	-	-	-	-
**Sun H et al. (35991920)**	-	-	-	-	-	-
**Rico-Jimenez JJ et al. (36307914)**	0.836	0.911	0.896			
**Lee J et al. (36354559)**	-	-	-	-	-	-
**Olender et al. et al. (35272217)**	0.806	0.779	-	0.806	0.779	-
**Lee J et al. (36465096)**	-	-	-	-		
**Lee J et al. (36509806)**	-	-	-	0.858	0.907	-
**Huang J et al. (36519717)**	-	-	-	-	-	-
**Chen T et al. (36434330)**	-	-	-	-	-	-
**Shi P et al. (36635865)**	-	-	-	-	-	-
**Fibrotic Tissue**						
**Ughi et al. (23847728)**	-	-	0.895	-	-	
**Athanasiou LS et al. (24109966)**	-	-	-	-	-	-
**Athanasiou LS et al. (24525828)**	0.87	-	-		-	-
**Celi S et al. (25077844)**	-	-	-	-	-	-
**Rico-Jimenez et al. (27867716)**	0.833	0.905	-	0.833	0.905	-
**Gnanadesigan M et al. (27998841)**	-	-	-	-	-	-
**Gnanadesigan et al. (27620900)**	-	-	-	-	-	-
**Liu et al. (28901053)**	-	-	-	-	-	-
**Zhou P et al. (29036125)**	-	-	-	-	-	
**Mengdi Xu et al. (29060164)**	-	-	-	-		
**Kolluru et al. (30525060)**	-	-	-			
**Gessert et al. (30130180)**	-	-	0.848			
**Liu R et al. (31535296)**	-	-	-	-	-	-
**Lee et al. (31853413)**	-	-	-	-	-	-
**Rico-Jimenez et al. (31604172)**		-	-	-		

Gharaibeh et al. (31903407	-	-	-	-	-	-
Athanasiou et al. (DOI: 10.1117/12.2513078)	-	-	0.963	-	-	
Yang et al. (https://doi.org/10.1016/j.bbe.2019.06.006)	-	-	0.94	-	-	
Min et al. (31718998)	-	-	-	-	-	-
Gerbaud E et al. (31326995)	-	-	-	-	-	-
Lee et al. (32054895)	-	-	-		-	-
He et al. (32914606)	-	-	-	-	-	-
Lee et al. (33598377)	-	-	-	-	-	-
Lee et al. (35291576)	-	-	-			
Chu et al. (33528359)	-	-	-	-	-	-
Abdolmanafi et al. (33914917)	0.94	0.99	0.96	0.94	0.99	0.96
Avital et al. (34050203)	-	-	-	-	-	-
Shibutani et al. (34126504)	-	-	-	-	-	-
Yin et al. (34222368)	0.95	0.97	-			
Isidori et al. (34292435)	-	-	-	-	-	-
Holmberg et al. (34970608)	-	-	-	-	-	-
Wu X et al. (35061247)	-	-	-	-		
Niioka H et al. (35982217)	-	-	-	-	-	-
Sun H et al. (35991920)	-	-	-	-	-	-
Rico-Jimenez JJ et al. (36307914)	-	-	-			
Lee J et al. (36354559)	-	-	-	-	-	-
Olender et al. et al. (35272217)	0.942	0.945	-	0.942	0.945	-
Lee J et al. (36465096)	-	-	-	-		
Lee J et al. (36509806)	-	-	-	-	-	-
Huang J et al. (36519717)	-	-	-	-	-	-
Chen T et al. (36434330)	-	-	-	-	-	-
Shi P et al. (36635865)	-	-	-	-	-	-

**Fibrous cap thickness/TCFA**						
**Ughi et al. (23847728)**	-	-	-	-	-	
**Athanasiou LS et al. (24109966)**	-	-	-	-	-	-
**Athanasiou LS et al. (24525828)**	-	-	-	-	-	-
**Celi S et al. (25077844)**	-	-	-	-	-	-
**Rico-Jimenez et al. (27867716)**	-	-	-	-	-	-
**Gnanadesigan M et al. (27998841)**	-	0.825	-	-	-	0.825
**Gnanadesigan et al. (27620900)**	-	-	-	-	-	-
**Liu et al. (28901053)**	-	-	-	-	-	-
**Zhou P et al. (29036125)**	-	-	-	-	-	
**Mengdi Xu et al. (29060164)**	-	-	-			-
**Kolluru et al. (30525060)**	-	-	-			
**Gessert et al. (30130180)**	-	-	-			
**Liu R et al. (31535296)**	-		0.808	-	0.8415	0.867
**Lee et al. (31853413)**	-	-	-	-	-	-
**Rico-Jimenez et al. (31604172)**		-	-	-		
**Gharaibeh et al. (31903407**	-	-	-	-	-	-
**Athanasiou et al. (DOI: 10.1117/12.2513078)**	-	-	-	-	-	
**Yang et al. (https://doi.org/10.1016/j.bbe.2019.06.006)**	-	-	-	-	-	
**Min et al. (31718998)**	0.887	0.918	0.916	0.886	0.932	0.928
**Gerbaud E et al. (31326995)**	-	-	-	-	-	-
**Lee et al. (32054895)**	-	-	-		-	-
**He et al. (32914606)**	-	-	-	-	-	-
**Lee et al. (33598377)**	-	-	-	-	-	-
**Lee et al. (35291576)**	-	-	-			
**Chu et al. (33528359)**	-	-	-	-	-	-
**Abdolmanafi et al. (33914917)**	-	-	-	-	-	-

**Avital et al. (34050203)**	-	-	-	-	-	-
**Shibutani et al. (34126504)**	-	-	-	-	-	-
**Yin et al. (34222368)**	-	-	-			
**Isidori et al. (34292435)**	-	-	-	-	-	-
**Holmberg et al. (34970608)**	-	-	-	-	-	-
**Wu X et al. (35061247)**	-	-	-	-		
**Niioka H et al. (35982217)**	-	0.940	-	-	-	0.94
**Sun H et al. (35991920)**	-	-	-	-	-	-
**Rico-Jimenez JJ et al. (36307914)**	-	-	-			
**Lee J et al. (36354559)**	-	-	-	-	-	-
**Olender et al. et al. (35272217)**	-	-	-	-	-	-
**Lee J et al. (36465096)**	-	-	-	-		
**Lee J et al. (36509806)**	-	-	-	-	-	-
**Huang J et al. (36519717)**	-	-	-	-	-	-
**Chen T et al. (36434330)**	-	-	-	-	-	-
**Shi P et al. (36635865)**	-	-	0.93	-	-	
**Plaque burden**						
**Ughi et al. (23847728)**	-	-	-	-	-	-
**Athanasiou LS et al. (24109966)**	-	-	-	-	-	-
**Athanasiou LS et al. (24525828)**	-	-	-	-	-	-
**Celi S et al. (25077844)**	-	-	-	-	-	-
**Rico-Jimenez et al. (27867716)**	-	-	-	-	-	-
**Gnanadesigan M et al. (27998841)**	-	-	-	-	-	-
**Gnanadesigan et al. (27620900)**	-	-	-	-	-	-
**Liu et al. (28901053)**	-	-	-	-	-	-
**Zhou P et al. (29036125)**	-	-	-	-	-	-
**Mengdi Xu et al. (29060164)**	-	-	-	-	-	-

**Kolluru et al. (30525060)**	-	-	-	-	-	-
**Gessert et al. (30130180)**	-	-	-	-	-	-
**Liu R et al. (31535296)**	-	-	-	-	-	-
**Lee et al. (31853413)**	-	-	-	-	-	-
**Rico-Jimenez et al. (31604172)**	-	-	-	-	-	-
**Gharaibeh et al. (31903407**	-	-	-	-	-	-
**Athanasiou et al. (DOI: 10.1117/12.2513078)**	-	-	-	-	-	-
**Yang et al. (https://doi.org/10.1016/j.bbe.2019.06.006)**	-	-	-	-	-	-
**Min et al. (31718998)**	-	-	-	-	-	-
**Gerbaud E et al. (31326995)**	-	-	-	-	-	-
**Lee et al. (32054895)**	-	-	-	-	-	-
**He et al. (32914606)**	-	-	-	-	-	-
**Lee et al. (33598377)**	-	-	-	-	-	-
**Lee et al. (35291576)**	-	-	-	-	-	-
**Chu et al. (33528359)**	-	-	-	-	-	-
**Abdolmanafi et al. (33914917)**	-	-	-	-	-	-
**Avital et al. (34050203)**	-	-	-	-	-	-
**Shibutani et al. (34126504)**	-	-	-	-	-	-
**Yin et al. (34222368)**	-	-	-	-	-	-
**Isidori et al. (34292435)**	-	-	-	-	-	-
**Holmberg et al. (34970608)**	-	-	-	-	-	-
**Wu X et al. (35061247)**	-	-	-	-	-	-
**Niioka H et al. (35982217)**	-	-	-	-	-	-
**Sun H et al. (35991920)**	-	-	-	-	-	-
**Rico-Jimenez JJ et al. (36307914)**	-	-	-	-	-	-
**Lee J et al. (36354559)**	-	-	-	-	-	-
**Olender et al. et al. (35272217)**	-	-	-	-	-	-

Lee J et al. (36465096)	-	-	-	-	-	-
Lee J et al. (36509806)	-	-	-	-	-	-
Huang J et al. (36519717)	-	-	0.92	-	-	-
Chen T et al. (36434330)	-	-	-	-	-	-
Shi P et al. (36635865)	-	-	-	-	-	-
**Pathological intimal thickening**						
Ughi et al. (23847728)	-	-	-	-	-	-
Athanasiou LS et al. (24109966)	-	-	-	-	-	-
Athanasiou LS et al. (24525828)	-	-	-	-	-	-
Celi S et al. (25077844)	-	-	-	-	-	-
Rico-Jimenez et al. (27867716)	-	-	-	-	-	-
Gnanadesigan M et al. (27998841)	-	-	-	-	-	-
Gnanadesigan et al. (27620900)	-	-	-	-	-	-
Liu et al. (28901053)	-	-	-	-	-	-
Zhou P et al. (29036125)	-	-	-	-	-	-
Mengdi Xu et al. (29060164)	-	-	-			-
Kolluru et al. (30525060)	-	-	-			-
Gessert et al. (30130180)	-	-	-			
Liu R et al. (31535296)	-	-	-	-	-	-
Lee et al. (31853413)	-	-	-	-	-	-
Rico-Jimenez et al. (31604172)	-	-	-			
Gharaibeh et al. (31903407	-	-	-	-	-	-
Athanasiou et al. (DOI: 10.1117/12.2513078)	-	-	-	-	-	-
Yang et al. (https://doi.org/10.1016/j.bbe.2019.06.006)	-	-	-	-	-	-
Min et al. (31718998)	-	-	-	-	-	-
Gerbaud E et al. (31326995)	-	-	-	-	-	-
Lee et al. (32054895)	-	-	-		-	-

**He et al. (32914606)**	-	-	-	-	-	-
**Lee et al. (33598377)**	-	-	-	-	-	-
**Lee et al. (35291576)**	-	-	-			
**Chu et al. (33528359)**	-	-	-	-	-	-
**Abdolmanafi et al. (33914917)**	-	-	-	-	-	-
**Avital et al. (34050203)**	-	-	-	-	-	-
**Shibutani et al. (34126504)**	-	-	-	0.74	0.85	0.85
**Yin et al. (34222368)**	-	-	-			
**Isidori et al. (34292435)**	-	-	-	-	-	-
**Holmberg et al. (34970608)**	-	-	-	-	-	-
**Wu X et al. (35061247)**	-	-	-			
**Niioka H et al. (35982217)**	-	-	-	-	-	-
**Sun H et al. (35991920)**	-	-	-	-	-	-
**Rico-Jimenez JJ et al. (36307914)**	-	-	-			
**Lee J et al. (36354559)**	-	-	-	-	-	-
**Olender et al. et al. (35272217)**	-	-	-	-	-	-
**Lee J et al. (36465096)**	-	-	-			
**Lee J et al. (36509806)**	-	-	-	-	-	-
**Huang J et al. (36519717)**	-	-	-	-	-	
**Chen T et al. (36434330)**	-	-	-	-	-	-
**Shi P et al. (36635865)**	-	-	-	-	-	-
**Neovascularization**						
**Ughi et al. (23847728)**	-	-	-	-	-	
**Athanasiou LS et al. (24109966)**	-	-	-	-	-	-
**Athanasiou LS et al. (24525828)**	-	-	-	-	-	-
**Celi S et al. (25077844)**	-	-	-	-	-	-
**Rico-Jimenez et al. (27867716)**	-	-	-	-	-	-

Gnanadesigan M et al. (27998841)	-	-	-	-	-	-
Gnanadesigan et al. (27620900)	-	-	-	-	-	-
Liu et al. (28901053)	-	-	-	-	-	-
Zhou P et al. (29036125)	-	-	-	-	-	
Mengdi Xu et al. (29060164)	-	-	-			-
Kolluru et al. (30525060)	-	-	-			
Gessert et al. (30130180)	-	-	-			
Liu R et al. (31535296)	-	-	-	-	-	-
Lee et al. (31853413)	-	-	-	-	-	-
Rico-Jimenez et al. (31604172)	-		-			
Gharaibeh et al. (31903407	-	-	-	-	-	-
Athanasiou et al. (DOI: 10.1117/12.2513078)	-	-	-	-	-	
Yang et al. (https://doi.org/10.1016/j.bbe.2019.06.006)	-	-	-	-	-	
Min et al. (31718998)	-	-	-	-	-	-
Gerbaud E et al. (31326995)	-	-	-	-	-	-
Lee et al. (32054895)	-	-	-		-	-
He et al. (32914606)	-	-	-	-	-	-
Lee et al. (33598377)	-	-	-	-	-	-
Lee et al. (35291576)	-	-	-			
Chu et al. (33528359)	-	-	-	-	-	-
Abdolmanafi et al. (33914917)	0.8	1	0.9	0.8	1	0.9
Avital et al. (34050203)	-	-	-	-	-	-
Shibutani et al. (34126504)	-	-	-	-	-	-
Yin et al. (34222368)	-	-	-			
Isidori et al. (34292435)	-	-	-	-	-	-
Holmberg et al. (34970608)	-	-	-	-	-	-
Wu X et al. (35061247)	0.644	-	-			

**Niioka H et al. (35982217)**	-	-	-	-	-	-
**Sun H et al. (35991920)**	-	-	-	-	-	-
**Rico-Jimenez JJ et al. (36307914)**	-	-	-			
**Lee J et al. (36354559)**	-	-	-	0.995	0.988	0.991
**Olender et al. et al. (35272217)**	-	-	-	-	-	-
**Lee J et al. (36465096)**	0.924	0.999	-	-		
**Lee J et al. (36509806)**	-	-	-	-	-	-
**Huang J et al. (36519717)**	-	-	-	-	-	-
**Chen T et al. (36434330)**	-	-	-	-	-	-
**Shi P et al. (36635865)**	-	-	-	-	-	-
**Macrophages**						
**Ughi et al. (23847728)**	-	-	-	-	-	
**Athanasiou LS et al. (24109966)**	-	-	-	-	-	-
**Athanasiou LS et al. (24525828)**	-	-	-	-	-	-
**Celi S et al. (25077844)**	-	-	-	-	-	-
**Rico-Jimenez et al. (27867716)**	-	-	-	-	-	-
**Gnanadesigan M et al. (27998841)**	-	-	-	-	-	-
**Gnanadesigan et al. (27620900)**	-	-	-	-	-	-
**Liu et al. (28901053)**	-	-	-	-	-	-
**Zhou P et al. (29036125)**	-	-	-	-	-	
**Mengdi Xu et al. (29060164)**	-	-	-			-
**Kolluru et al. (30525060)**	-	-	-			
**Gessert et al. (30130180)**	-	-	-			
**Liu R et al. (31535296)**	-	-	-	-	-	-
**Lee et al. (31853413)**	-	-	-	-	-	-
**Rico-Jimenez et al. (31604172)**	0.845	0.892	0.882			
**Gharaibeh et al. (31903407**	-	-	-	-	-	-

**Athanasiou et al. (DOI: 10.1117/12.2513078)**	-	-	-	-	-	
**Yang et al. (https://doi.org/10.1016/j.bbe.2019.06.006)**	-	-	-	-	-	
**Min et al. (31718998)**	-	-	-	-	-	-
**Gerbaud E et al. (31326995)**	-	-	-	-	-	-
**Lee et al. (32054895)**	-	-	-		-	-
**He et al. (32914606)**	-	-	-	-	-	-
**Lee et al. (33598377)**	-	-	-	-	-	-
**Lee et al. (35291576)**	-	-	-			
**Chu et al. (33528359)**	-	-	-	-	-	-
**Abdolmanafi et al. (33914917)**	-	-	-	-	-	-
**Avital et al. (34050203)**	-	-	-	-	-	-
**Shibutani et al. (34126504)**	-	-	-	-	-	-
**Yin et al. (34222368)**	-	-	-			
**Isidori et al. (34292435)**	-	-	-	-	-	-
**Holmberg et al. (34970608)**	-	-	-	-	-	-
**Wu X et al. (35061247)**	-	-	-	-		
**Niioka H et al. (35982217)**	-	-	-	-	-	-
**Sun H et al. (35991920)**	-	-	-	-	-	-
**Rico-Jimenez JJ et al. (36307914)**	-	-	-			
**Lee J et al. (36354559)**	-	-	-	-	-	-
**Olender et al. et al. (35272217)**	-	-	-	-	-	-
**Lee J et al. (36465096)**	-	-	-	-		
**Lee J et al. (36509806)**	-	-	-	-	-	-
**Huang J et al. (36519717)**	-	-	-	-	-	-
**Chen T et al. (36434330)**	-	-	-	-	-	-
**Shi P et al. (36635865)**	-	-	-	-	-	-
**Calcified nodules**						

Ughi et al. (23847728)	-	-	-	-	-	-
Athanasiou LS et al. (24109966)	-	-	-	-	-	-
Athanasiou LS et al. (24525828)	-	-	-		-	-
Celi S et al. (25077844)	-	-	-	-	-	-
Rico-Jimenez et al. (27867716)	-	-	-	-	-	-
Gnanadesigan M et al. (27998841)	-	-	-	-	-	-
Gnanadesigan et al. (27620900)	-	-	-	-	-	-
Liu et al. (28901053)	-	-	-	-	-	-
Zhou P et al. (29036125)	-	-	-	-	-	
Mengdi Xu et al. (29060164)	-	-	-	-		
Kolluru et al. (30525060)	-	-	-			
Gessert et al. (30130180)	-	-	-			
Liu R et al. (31535296)	-	-	-	-	-	-
Lee et al. (31853413)	-	-	-	-	-	-
Rico-Jimenez et al. (31604172)		-	-	-		
Gharaibeh et al. (31903407	-	-	-	-	-	-
Athanasiou et al. (DOI: 10.1117/12.2513078)	-	-	-	-	-	
Yang et al. (https://doi.org/10.1016/j.bbe.2019.06.006)	-	-	-	-	-	
Min et al. (31718998)	-	-	-	-	-	-
Gerbaud E et al. (31326995)	-	-	-	-	-	-
Lee et al. (32054895)	-	-	-			-
He et al. (32914606)	-	-	-	-	-	-
Lee et al. (33598377)	-	-	-	-	-	-
Lee et al. (35291576)	-	-	-			
Chu et al. (33528359)	-	-	-	-	-	-
Abdolmanafi et al. (33914917)	-	-	-	-	-	-
Avital et al. (34050203)	-	-	-	-	-	-

**Shibutani et al. (34126504)**	-	-	-	-	-	-
**Yin et al. (34222368)**	-	-	-			
**Isidori et al. (34292435)**	-	-	-	-	-	-
**Holmberg et al. (34970608)**	-	-	-	-	-	-
**Wu X et al. (35061247)**	-	-	-	-		
**Niioka H et al. (35982217)**	-	-	-	-	-	-
**Sun H et al. (35991920)**	-	-	-	-	-	-
**Rico-Jimenez JJ et al. (36307914)**	-	-	-	-		
**Lee J et al. (36354559)**	-	-	-	-	-	-
**Olender et al. et al. (35272217)**	-	-	-	-	-	-
**Lee J et al. (36465096)**	-	-	-	-		
**Lee J et al. (36509806)**	-	-	-	-	-	-
**Huang J et al. (36519717)**	-	-	-	-	-	-
**Chen T et al. (36434330)**	0.917	0.893	0.91	0.917	0.893	0.91
**Shi P et al. (36635865)**	-	-	-	-	-	-
**Cholesterol crystals**						
**Ughi et al. (23847728)**	-	-	-	-	-	
**Athanasiou LS et al. (24109966)**	-	-	-	-	-	-
**Athanasiou LS et al. (24525828)**	-	-	-	-	-	-
**Celi S et al. (25077844)**	-	-	-	-	-	-
**Rico-Jimenez et al. (27867716)**	-	-	-	-	-	-
**Gnanadesigan M et al. (27998841)**	-	-	-	-	-	-
**Gnanadesigan et al. (27620900)**	-	-	-	-	-	-
**Liu et al. (28901053)**	-	-	-	-	-	-
**Zhou P et al. (29036125)**	-	-	-	-	-	
**Mengdi Xu et al. (29060164)**	-	-	-			-
**Kolluru et al. (30525060)**	-	-	-			

Gessert et al. (30130180)	-	-	-			
Liu R et al. (31535296)	-	-	-	-	-	-
Lee et al. (31853413)	-	-	-	-	-	-
Rico-Jimenez et al. (31604172)	-	-	-	-	-	-
Gharaibeh et al. (31903407	-	-	-	-	-	-
Athanasiou et al. (DOI: 10.1117/12.2513078)	-	-	-	-	-	-
Yang et al. (https://doi.org/10.1016/j.bbe.2019.06.006)	-	-	-	-	-	-
Min et al. (31718998)	-	-	-	-	-	-
Gerbaud E et al. (31326995)	-	-	-	-	-	-
Lee et al. (32054895)	-	-	-		-	-
He et al. (32914606)	-	-	-	-	-	-
Lee et al. (33598377)	-	-	-	-	-	-
Lee et al. (35291576)	-	-	-	-	-	-
Chu et al. (33528359)	-	-	-	-	-	-
Abdolmanafi et al. (33914917)	-	-	-	-	-	-
Avital et al. (34050203)	-	-	-	-	-	-
Shibutani et al. (34126504)	-	-	-	-	-	-
Yin et al. (34222368)	-	-	-			
Isidori et al. (34292435)	-	-	-	-	-	-
Holmberg et al. (34970608)	-	-	-	-	-	-
Wu X et al. (35061247)	-	-	-	-		
Niioka H et al. (35982217)	-	-	-	-	-	-
Sun H et al. (35991920)	-	-	-	-	-	-
Rico-Jimenez JJ et al. (36307914)	-	-	-			
Lee J et al. (36354559)	-	-	-	-	-	-
Olender et al. et al. (35272217)	-	-	-	-	-	-
Lee J et al. (36465096)	-	-	-	-		

Lee J et al. (36509806)	-	-	-	-	-	-
Huang J et al. (36519717)	-	-	-	-	-	-
Chen T et al. (36434330)	-	-	-	-	-	-
Shi P et al. (36635865)	-	-	-	-	-	-
Microchannels						
Ughi et al. (23847728)	-	-	-	-	-	
Athanasiou LS et al. (24109966)	-	-	-	-	-	-
Athanasiou LS et al. (24525828)	-	-	-	-	-	-
Celi S et al. (25077844)	-	-	-	-	-	-
Rico-Jimenez et al. (27867716)	-	-	-	-	-	-
Gnanadesigan M et al. (27998841)	-	-	-	-	-	-
Gnanadesigan et al. (27620900)	-	-	-	-	-	-
Liu et al. (28901053)	-	-	-	-	-	-
Zhou P et al. (29036125)	-	-	-	-	-	
Mengdi Xu et al. (29060164)	-	-	-			-
Kolluru et al. (30525060)	-	-	-			
Gessert et al. (30130180)	-	-	-			
Liu R et al. (31535296)	-	-	-	-	-	-
Lee et al. (31853413)	-	-	-	-	-	-
Rico-Jimenez et al. (31604172)	-	-	-	-	-	-
Gharaibeh et al. (31903407	-	-	-	-	-	-
Athanasiou et al. (DOI: 10.1117/12.2513078)	-	-	-	-	-	-
Yang et al. (https://doi.org/10.1016/j.bbe.2019.06.006)	-	-	-	-	-	-
Min et al. (31718998)	-	-	-	-	-	-
Gerbaud E et al. (31326995)	-	-	-	-	-	-
Lee et al. (32054895)	-	-	-		-	-
He et al. (32914606)	-	-	-	-	-	-

**Lee et al. (33598377)**	-	-	-	-	-	-
**Lee et al. (35291576)**	-	-	-	-	-	-
**Chu et al. (33528359)**	-	-	-	-	-	-
**Abdolmanafi et al. (33914917)**	-	-	-	-	-	-
**Avital et al. (34050203)**	-	-	-	-	-	-
**Shibutani et al. (34126504)**	-	-	-	-	-	-
**Yin et al. (34222368)**	-	-	-			
**Isidori et al. (34292435)**	-	-	-	-	-	-
**Holmberg et al. (34970608)**	-	-	-	-	-	-
**Wu X et al. (35061247)**	-	-	-	-		
**Niioka H et al. (35982217)**	-	-	-	-	-	-
**Sun H et al. (35991920)**	-	-	-	-	-	-
**Rico-Jimenez JJ et al. (36307914)**	-	-	-			
**Lee J et al. (36354559)**	-	-	-	-	-	-
**Olender et al. et al. (35272217)**	-	-	-	-	-	-
**Lee J et al. (36465096)**	-	-	-	-		
**Lee J et al. (36509806)**	-	-	-	-	-	-
**Huang J et al. (36519717)**	-	-	-	-	-	-
**Chen T et al. (36434330)**	-	-	-	-	-	-
**Shi P et al. (36635865)**	-	-	-	-	-	-

-

Supplementary table 4. Quantitative metrics for diagnostic performance of automatic IVUS tools.

First Author	Training set			Testing set		
	Sensitivity	Specificity	Diagnostic Accuracy	Sensitivity	Specificity	Diagnostic accuracy
**Calcium**						
**Zhang Q et al. (19900745)**	0.871	0.873	0.907			
**Vard A et al. (22415899)**	.	.	.	.	.	.
**Gao et al. (25372784)**	0.947	0.958	.			.
**Gao Z et al. (25922134)**	.	.	.	.	.	.
**Araki T et al. (26643081)**	.	.	.	.	.	
**Banchhor SK et al. (27480747)**	.	.	.	.	.	.
**Lo Vercio L et al. (26811082)**	.	.	.	.	.	.
**Banchhor SK et al. (28390284)**	.	.	.	.	.	.
**Su S et al. (28062170)**	.	.	.	.	.	.
**Zakeri FS et al. (28372789)**	.	.	.	.	.	.
**Faraji M et al. (29241056)**	.	.	.	.	.	.
**Bae Y et al. (31130215)**	.	.	.	.	.	.
**Jun TJ et al. (30426362)**	.	.	.	.	.	.
**Lo Vercio L et al. (31319939)**	.	.	.	.	.	.
**Huang Y et al. (31978806)**	.	.	.	.	.	.
**Liu S et al. (32636052)**	.	.	Infraredx 40Mhz: 0.917, Volcano 20 Mhz: 0.911, Boston Scientific 40Mhz: 0.908	.	.	Infraredx 40Mhz: 0.87, Volcano 20 Mhz: 0.89, Boston Scientific 40Mhz 0.89
**Li et al. (34351974)**	.	.	.	.		0.98
**Shinohara et al. (33460377)**	.	.	.			.

Bajaj R et al. (34153412)	.	.	.	.	.	.
Cho H et al. (33831671)	.	.	.	0.86	0.97	0.96
Dong L et al. (33549115)	.	.	.	.	.	.
Nishi T et al. (33741429)	.	.	.	.	.	.
Bajaj et al. (35196627)	.	.	.	.	.	.
Zhu et al. (35861418)	.	.	.	.	.	.
Blanco PJ et al. (34670148)	.	.	.	.	.	.
Du H et al. (34974233)	.	.	.	.	.	.
**Plaque burden**						
Zhang Q et al. (19900745)	.	.	.			.
Vard A et al. (22415899)	.	.	.	.	.	.
Gao et al. (25372784)	.	.	.		.	.
Gao Z et al. (25922134)	.	.	.	.	.	.
Araki T et al. (26643081)	.	.	.	.	.	.
Banchhor SK et al. (27480747)	.	.	.	.	.	.
Lo Vercio L et al. (26811082)	.	.	.	.	.	.
Banchhor SK et al. (28390284)	.	.	.	.	.	.
Su S et al. (28062170)	.	.	.	.	.	.
Zakeri FS et al. (28372789)	.	.	.	.	.	.
Faraji M et al. (29241056)	.	.	.	.	.	.
Bae Y et al. (31130215)	.	.	.	.	.	0.702
Jun TJ et al. (30426362)	.	.	.	.	.	.
Lo Vercio L et al. (31319939)	.	.	.	.	.	.
Huang Y et al. (31978806)	.	.	.	.	.	.
Liu S et al. (32636052)	.	.	.	.	.	.
Li et al. (34351974)	.	.	.	.	.	.
Shinohara et al. (33460377)	.	.	.	.	.	.
Bajaj R et al. (34153412)	.	.	.	.	.	.

**Cho H et al. (33831671)**	.	.	.	.	.	.
**Dong L et al. (33549115)**	.	.	.	.	.	.
**Nishi T et al. (33741429)**	.	.	.	.	.	.
**Bajaj et al. (35196627)**	.	.	.	.	.	.
**Zhu et al. (35861418)**	.	.	.	.	.	.
**Blanco PJ et al. (34670148)**	.	.	.	.	.	.
**Du H et al. (34974233)**	.	.	.	.	.	.
**Fibrous cap thickness/TCFA**						
**Zhang Q et al. (19900745)**	.	.	.			.
**Vard A et al. (22415899)**	.	.	.	.	.	.
**Gao et al. (25372784)**	.	.	.		.	.
**Gao Z et al. (25922134)**	.	.	.	.	.	.
**Araki T et al. (26643081)**	.	.	.	.	.	.
**Banchhor SK et al. (27480747)**	.	.	.	.	.	.
**Lo Vercio L et al. (26811082)**	.	.	.	.	.	.
**Banchhor SK et al. (28390284)**	.	.	.	.	.	.
**Su S et al. (28062170)**	.	.	.	.	.	.
**Zakeri FS et al. (28372789)**	.	.	.	.	.	.
**Faraji M et al. (29241056)**	.	.	.	.	.	.
**Bae Y et al. (31130215)**	0.85	0.79	0.8	0.81	0.79	0.82
**Jun TJ et al. (30426362)**	.	.	.	0.8731	0.8281	0.911
**Lo Vercio L et al. (31319939)**	.	.	.	.	.	.
**Huang Y et al. (31978806)**	.	.	.	.	.	.
**Liu S et al. (32636052)**	.	.	.	.	.	.
**Li et al. (34351974)**	.	.	.	.	.	.
**Shinohara et al. (33460377)**	.	.	.	.	.	.
**Bajaj R et al. (34153412)**	.	.	.	.	.	.
**Cho H et al. (33831671)**	.	.	.	.	.	.

**Dong L et al. (33549115)**	.	.	.	.	.	.
**Nishi T et al. (33741429)**	.	.	.	.	.	.
**Bajaj et al. (35196627)**	.	.	.	.	.	.
**Zhu et al. (35861418)**	.	.	.	.	.	.
**Blanco PJ et al. (34670148)**	.	.	.	.	.	.
**Du H et al. (34974233)**	.	.	.	.	.	.
**Fibrotic tissue**						
**Zhang Q et al. (19900745)**	.	.	.	.	.	.
**Vard A et al. (22415899)**	.	.	.	.	.	.
**Gao et al. (25372784)**	.	.	.	.	.	.
**Gao Z et al. (25922134)**	.	.	.	.	.	.
**Araki T et al. (26643081)**	.	.	.	.	.	.
**Banchhor SK et al. (27480747)**	.	.	.	.	.	.
**Lo Vercio L et al. (26811082)**	.	.	.	.	.	.
**Banchhor SK et al. (28390284)**	.	.	.	.	.	.
**Su S et al. (28062170)**	.	.	.	.	.	.
**Zakeri FS et al. (28372789)**	.	.	.	.	.	.
**Faraji M et al. (29241056)**	.	.	.	.	.	.
**Bae Y et al. (31130215)**	-	.	.	.	.	.
**Jun TJ et al. (30426362)**	.	.	.	.	.	.
**Lo Vercio L et al. (31319939)**	.	.	.	.	.	.
**Huang Y et al. (31978806)**	.	.	.	.	.	.
**Liu S et al. (32636052)**	.	.	.	.	.	.
**Li et al. (34351974)**	.	.	.	.	.	.
**Shinohara et al. (33460377)**	.	.	.	.	.	.
**Bajaj R et al. (34153412)**	.	.	.	.	.	.
**Cho H et al. (33831671)**	.	.	.	.	.	.
**Dong L et al. (33549115)**	.	.	.	.	.	.

**Nishi T et al. (33741429)**	.	.	.	.	.	.
**Bajaj et al. (35196627)**	.	.	.	.	.	.
**Zhu et al. (35861418)**	.	.	.	.	.	.
**Blanco PJ et al. (34670148)**	.	.	.	.	.	.
**Du H et al. (34974233)**	.	.	.	.	.	.
